# Physical Activity Determinants Under the Double Burden of Malnutrition: Contrasting Pathways for Underweight and Overweight Chinese Adolescents

**DOI:** 10.3390/nu18010179

**Published:** 2026-01-05

**Authors:** Liying Yao, Shuaishuai Jia, Xiaochang Lv, Yongguan Dai, Yee Cheng Kueh, Jinfu Xu, Jianqiu Cong, Garry Kuan

**Affiliations:** 1 School of Physical Education and Sport Science, Guangzhou University, Guangzhou 510006, China; yaoliying@gzhu.edu.cn (L.Y.); xiaochang831@163.com (X.L.); daiyongguan1976@163.com (Y.D.); 2 Exercise and Sports Science Programme, School of Health Sciences, Universiti Sains Malaysia, Kubang Kerian 16150, Kelantan, Malaysia; 3 Guangzhou Institute of International Finance, Guangzhou University, Guangzhou 510006, China; 4 Biostatistics and Research Methodology Unit, School of Medical Sciences, Universiti Sains Malaysia, Kubang Kerian 16150, Kelantan, Malaysia; yckueh@usm.my; 5 Journal Center, Guangzhou Sport University, Guangzhou 510500, China; 11526@gzsport.edu.cn; 6 College of Physical Education, Kaili University, Kaili 556011, China; congjianqiu@163.com

**Keywords:** body mass index, multi-group structural equation modeling, precision public health, Social-Ecological Model, Transtheoretical Model

## Abstract

Background: Chinese adolescents face a dual burden of malnutrition, yet the weight-status-specific mechanisms underlying physical activity (PA) participation remain underexplored. Methods: We conducted a cross-sectional study among 1573 adolescents (aged 9–15 years) in Shangrao City, China. Validated scales measured social-ecological factors (family/peer support, physical environment), psychological factors (stage of change, self-efficacy, decisional balance), and PA participation. Data preprocessing utilized full information maximum likelihood to handle missing values. Confirmatory factor analysis was performed to validate the measurement model, followed by multi-group structural equation modeling to analyze pathway configurations across underweight (*n* = 187), normal-weight (*n* = 1070), and overweight/obese (*n* = 316) groups. Mediation effects were tested using bootstrapping with 5000 resamples. Results: Clear weight-specific patterns emerged. Normal-weight adolescents presented a fully functional comprehensive model where PA was predicted by the stage of change (β = 0.211, *p* < 0.001), friend support (β = 0.120, *p* < 0.001), self-efficacy (β = 0.092, *p* < 0.05), and perceived benefits (β = 0.095, *p* < 0.01). Underweight adolescents primarily relied on internal readiness driven by stage of change (β = 0.270, *p* < 0.001) and self-efficacy (β = 0.164, *p* < 0.05), with family support only indirectly influencing participation via psychological mediators. In contrast, overweight/obese adolescents showed a “socially dependent” pattern: friend support directly predicted PA levels (β = 0.136, *p* < 0.05), significantly enhanced self-efficacy (β = 0.370, *p* < 0.01), and effectively lowered perceived barriers (β = −0.165, *p* < 0.05). Additionally, the physical environment strongly impacted perceived benefits (β = 0.471, *p* < 0.01) but did not translate into action. Conclusions: These findings underscore the significant differences in PA determinants across the spectrum of malnutrition, necessitating targeted public health interventions to support the Healthy China 2030 initiative.

## 1. Introduction

China is currently undergoing a rapid nutritional transition characterized by a “double burden” of malnutrition among its youth [1,2]. While the rapid rise in childhood overweight and obesity has reached epidemic proportions, a significant proportion of adolescents remain underweight [3,4]. The latest official data reveals that while nearly 20% of Chinese school-aged children are overweight or obese [5], the prevalence of underweight remains high in specific sociodemographic groups, posing parallel risks to health and development [6]. This BMI polarization presents complex public health challenges: both extremes of weight distribution correlate with adverse metabolic indicators and psychological distress. Critically, both groups exhibit insufficient physical activity (PA) behavior [7,8]. Addressing this dual challenge requires a more comprehensive understanding of health behaviors across the entire BMI spectrum.

PA is a fundamental determinant of energy balance and the cornerstone for managing weight-related health risks [9,10]. However, PA levels among Chinese adolescents are showing a concerning downward trend. Less than 30% of adolescents meet the World Health Organization’s recommendation of 60 min of moderate-to-vigorous physical activity (MVPA) daily [11]. This physical inactivity crisis is deeply associated with China’s unique sociocultural structure. Deeply ingrained Confucian values that prioritize academic achievement, coupled with intense academic competition, often relegate physical health to secondary consideration, creating structural barriers that limit leisure time and space [12]. Furthermore, within the collectivist context, peer acceptance and high levels of parental involvement exert significant influence on shaping adolescent behavior [13,14].

Crucially, however, these broad cultural forces likely interact with individual weight status in complex and distinct ways [15,16]. For overweight and obese adolescents, physical inactivity is often driven by a vicious cycle of specific “weight-related barriers” [17]. Recent research indicates that body dissatisfaction and fear of stigmatization significantly inhibit their willingness to engage in MVPA [18,19]. Consequently, this group may require more robust social support frameworks to overcome these specific psychological barriers, rather than generic encouragement.

In contrast, the determinants of PA among underweight adolescents are much less understood. Unlike their normal-weight peers, underweight youth often lack the motivation to exercise for weight control, creating a “sedentary but thin” risk profile that masks underlying health issues [20,21]. Emerging evidence suggests their activity levels may be driven less by weight goals and more by family health beliefs, yet few studies have empirically compared these pathways with those of overweight groups.

However, most current research still relies primarily on one-size-fits-all models that assume factors such as peer support exert uniform effects on all adolescents. It remains unclear whether the protective effects of social support for stigmatized obese children are equivalent to those observed in underweight adolescents who perceive themselves as “naturally thin” and thus see no need for exercise. Therefore, further investigation is needed into how these socio-ecological forces interact with psychological readiness to influence PA behavior differently among adolescents across the BMI spectrum.

**Figure 1:**
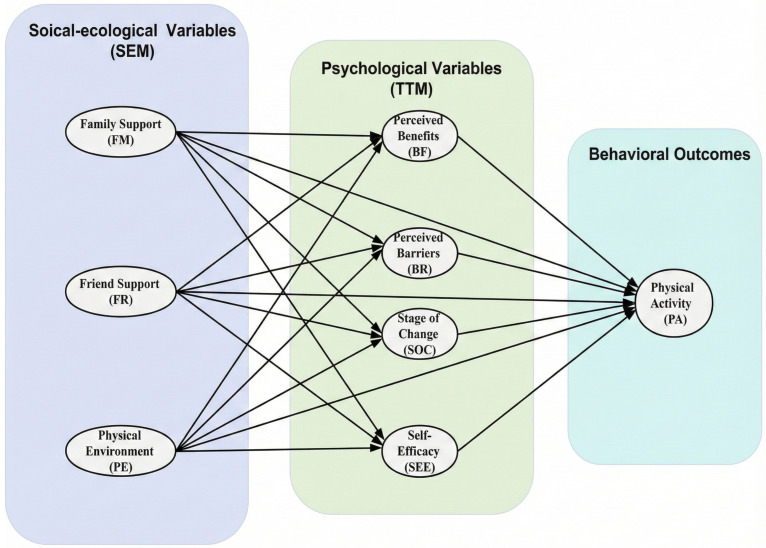
Conceptual model of the relationships between social-ecological variables, psychological factors, and PA.

To address these important knowledge gaps, this study constructs an integrated theoretical framework that combines the Social Ecological Model (SEM) and the Transtheoretical Model (TTM). The Social Ecological Model highlights the influence of interpersonal environments (family and friend support) and physical environmental factors [22,23,24]. At the same time, the Transtheoretical Model explains the psychological mechanisms driving behavioral implementation, specifically including self-efficacy, decisional balance, and stages of change [25]. By integrating these perspectives (see Figure 1), we move beyond examining isolated predictors to examine how socioecological factors directly or indirectly influence PA among adolescents with different BMIs in China, with psychological variables mediating these effects. Specifically, this study employs a multi-group structural equation model to analyze the heterogeneity of these pathways across three distinct BMI groups: underweight, normal weight, and overweight or obese. Based on this framework, we propose two core hypotheses:

**Hypothesis 1** **(H1):**
*Adolescents in different weight categories will demonstrate significant differences in the levels of PA and associated psychosocial determinants (e.g., self-efficacy, perceived barriers).*

**Hypothesis 2** **(H2):**
*The structural mechanisms predicting PA will vary by weight status. Specifically, we hypothesize that social support will be a dominant predictor for overweight/obese adolescents (acting as a buffer against barriers), whereas underweight adolescents will rely more heavily on family influence and internal readiness.*

## 2. Materials and Methods

### 2.1. Study Design

This cross-sectional study examined relationships among social-ecological factors (SEM framework), psychological factors (TTM framework), and PA levels across BMI categories in Chinese youth aged 9–15 years. The integrated theoretical approach enabled assessment of environmental factors (family support, friend support, physical environment) and psychological determinants (stages of change, self-efficacy, decisional balance) of PA behavior. Data were collected from November 2021 to March 2022 in Shangrao City, Jiangxi Province, using multistage cluster sampling across urban and suburban schools.

### 2.2. Participants

A total of 1573 children and adolescents participated in the study. The multistage cluster sampling involved: (1) stratifying Shangrao City into urban and suburban districts based on administrative boundaries and socioeconomic characteristics; (2) randomly selecting two primary schools and two middle schools from each stratum using computer-generated randomization; and (3) randomly selecting five classes spanning grades 4–9 from each school, with all students in selected classes invited to participate.

Inclusion criteria comprised: (a) Chinese nationality; (b) ability to comprehend study materials and provide informed assent; and (c) absence of medical contraindications to PA. Exclusion criteria included: (a) diagnosed physical disabilities limiting PA participation; (b) cognitive impairments affecting questionnaire completion; and (c) chronic medical conditions requiring activity restriction as verified by school health records.

### 2.3. Sample Size Calculation

Sample size was calculated following SEM guidelines, which recommend 10–20 participants per estimated parameter [26]. For multi-group analyses, simulation studies indicate that group sizes of 100–150 can produce reliable estimates when models have strong factor loadings [27]. The achieved sample of 1573 participants exceeded requirements for adequate statistical power (0.80) to detect medium effect sizes at α = 0.05. Maximum likelihood estimation with robust standard errors (MLR) was employed to optimize parameter estimation across groups.

### 2.4. Anthropometry Technique and Nutritional Status

Trained research staff measured height and weight following standardized protocols [11]. Standing height was measured to the nearest 0.1 cm using a portable stadiometer with participants barefoot. Weight was assessed to the nearest 0.1 kg using a calibrated digital scale with participants in light clothing. BMI was calculated as weight (kg) divided by height squared (m^2^). To investigate the double burden of malnutrition, nutritional status was classified using the age- and sex-specific BMI cut-offs recommended by the Working Group on Obesity in China (2018) standards [28]. Consistent with WHO conceptualizations [3], malnutrition was operationalized as encompassing both undernutrition (defined as underweight status) and overnutrition (defined as overweight and obese status). This categorization enables researchers to examine coexisting health burdens within participants and to analyze specific PA pathways across different nutritional statuses.

### 2.5. Measurement Tools

All instruments underwent forward-backward translation, expert panel review, and pilot testing with 120 students.

1. Chinese Version of Social Support Scale for Exercise (SE-C): Originally developed by Sallis et al. [24] and adapted by Yang et al. [29], this 24-item scale measures family support (12 items) and friend support (12 items) for PA. Items are rated on a 5-point scale (1 = never to 5 = very often). The Chinese version was translated and validated for this study [30]. Reliability: α = 0.966.

2. Chinese Version of Physical Environment Scale for Exercise (PE-C): Developed by Stahl et al. [31], this 5-item scale assesses availability (3 items) and quality (2 items) of PA facilities. The scale was translated and validated for Chinese youth in this study using standard forward-backward translation procedures. Reliability: α = 0.886.

3. Chinese Version of Decisional Balance Scale (DB-C): This 10-item scale [32] measures perceived benefits and barriers to PA on a 5-point importance scale. While a Chinese version exists for Taiwan [33], this study developed and validated a mainland China version. Reliability: α = 0.809.

4. Chinese Version of Self-Efficacy for Exercise Scale (SEE-C): Based on Bandura’s [34] 18-item scale assessing confidence in maintaining regular exercise. Participants rate confidence on a 5-point scale (1 = not at all confident to 5 = completely confident). The Chinese version was developed and validated for this study [35]. Reliability: α = 0.964.

5. Chinese Version of Stage of Change Scale (SOC-C): A single-item measure categorizing exercise behavior into six stages: precontemplation, contemplation, preparation, action, maintenance, and relapse [36]. The Chinese version was translated and validated by a panel of experts in health psychology and sports science [37].

6. Chinese Version of International Physical Activity Questionnaire (IPAQ-C): The 7-item Chinese version assesses PA intensity and sitting time over the past seven days. Total PA is calculated as MET-minutes/week. The Chinese version demonstrated acceptable reliability (intraclass correlation coefficient, ICC > 0.70) and validity in previous studies [38].

### 2.6. Data Collection

Data were collected between November 2021 and March 2022 using self-administered questionnaires. Each questionnaire included socio-demographic information and scales measuring social-ecological variables, psychological factors, and PA levels. Participants completed the questionnaires in approximately 15–25 min. Promotional posters were displayed to encourage participation, and parents provided informed consent for their children’s involvement.

### 2.7. Statistical Analysis

Data analyses were performed using SPSS 28.0 and Mplus 8.7 [39,40]. Descriptive statistics were calculated for all variables. Missing data (<5%) were handled using full information maximum likelihood. Multi-group structural equation modeling examined relationships among social-ecological variables, TTM variables, and PA across BMI categories (underweight, normal weight, overweight/obese). The analysis involved: (1) measurement model validation through confirmatory factor analysis; (2) structural model testing with social-ecological variables as predictors, TTM constructs as mediators, and PA as outcome; and (3) multi-group invariance testing. Model fit was evaluated using the root mean square error of approximation (RMSEA) < 0.05, comparative fit index (CFI) > 0.92, Tucker–Lewis index (TLI) > 0.92, and standardized root mean square residual (SRMR) < 0.08 [16]. Indirect effects were tested using bootstrapping with 5000 resamples. Statistical significance was set at *p* < 0.05 [40].

### 2.8. Ethical Consideration

Ethical approval was obtained from the Human Ethics Committee of Universiti Sains Malaysia (JEPeM Code: USM/JEPeM/21090638) and Jiangxi Medical College (Approval No: (RH)2022-5). The study adhered to the principles of the Declaration of Helsinki. Participants were informed about the study’s purpose, procedures, and confidentiality measures and provided written informed consent. Consent was also obtained from the original authors of the questionnaires.

## 3. Results

### 3.1. Participant Characteristics

The final sample consisted of 1573 adolescents (57% boys) with a mean age of 12.0 years (standard deviation [SD] = 1.68). According to BMI classification, 187 participants (11.9%) were underweight, 1070 (68%) were of normal weight, and 316 (20.1%) were overweight and obese. On average, participants engaged in PA 4.0 times per week (SD = 2.52), with each session lasting approximately 36.1 min (SD = 31.1). Regarding PA levels, 19.5% of students met the criteria for high activity, 59.2% were at a moderate level, and 21.3% fell into the low activity category (see Table 1).

### 3.2. Preliminary Analyses

#### 3.2.1. Assumption Testing

Multivariate normality was assessed using Mardia’s test, which indicated significant departure from normality (*p* < 0.001), necessitating the use of maximum likelihood with robust standard errors (MLR). Multicollinearity diagnostics revealed no issues, with all variance inflation factors below 5.0.

#### 3.2.2. Measurement Model Testing

Confirmatory factor analysis validated all the measurement scales that hadn’t been validated. Factor loadings ranged from 0.68 to 0.91, with composite reliabilities exceeding 0.80 for all constructs (see Table 2).

### 3.3. Structural Equation Modeling

#### Full Sample Model

The initial structural model examining relationships among social-ecological variables (family support, friend support, physical environment), TTM constructs (SOC, decisional balance, self-efficacy), and PA yielded suboptimal fit: χ^2^ (1632) = 6089.291, *p* < 0.001, χ^2^/*df* = 3.73, RMSEA = 0.042, CFI = 0.907, TLI = 0.903, SRMR = 0.050. Of the 19 hypothesized paths, four were non-significant: family support → barriers (β = −0.035, *p* = 0.424), family support → PA (β = −0.007, *p* = 0.856), physical environment → PA (β = 0.038, *p* = 0.193), and barriers → PA (β = −0.046, *p* = 0.067).

Model modification involved removing non-significant paths and adding four theoretically justified covariances (SEE7 with SEE6; SEE6 with SEE5; SEE8 with SEE4; FM4 with FM1). The modified model achieved acceptable fit: χ^2^ (1631) = 5233.309, *p* < 0.001, χ^2^/*df* = 3.20, RMSEA = 0.037 (90% CI [0.036, 0.039]), CFI = 0.925, TLI = 0.921, SRMR = 0.049. Fifteen of 19 hypothesized paths were supported (see Table 3).

Table 4 presents the standardized path coefficients for the final structural model across the full sample. Consistent with our theoretical expectations, social-ecological factors demonstrated distinct associations with psychological factors. Physical environment emerged as the strongest predictor of perceived benefits (β = 0.349, *p* < 0.001), while friend support, as a key resource, significantly predicted self-efficacy (β = 0.343, *p* < 0.001) and effectively reduced perceived barriers (β = −0.168, *p* < 0.001). Although family support positively correlated with all psychological mediating variables, its effect size was smaller than that of friend support and environmental factors. Regarding direct determinants of PA, stage of change (β = 0.219, *p* < 0.001) and friend support (β = 0.120, *p* < 0.001) emerged as the strongest predictors. Perceived benefits (β = 0.089, *p* = 0.004), and self-efficacy (β = 0.104, *p* = 0.001) also exhibited significant positive effects. Notably, neither family support nor physical environment exerted a significant direct influence on PA levels in the full sample, suggesting their effects may be fully mediated or exhibit subgroup differences—a possibility to be verified through subsequent multigroup analyses.

### 3.4. Multi-Group Analysis

#### 3.4.1. Measurement Invariance

Measure invariance testing was conducted as a precondition for valid multi-group comparisons, aiming to ensure equivalence in construct interpretation across BMI categories. The configuration model (without equality constraints) demonstrated acceptable fit (CFI = 0.925), thereby establishing a baseline for the comparable factor structure. Subsequent tests supported measurement invariance, with negligible fit decline (ΔCFI = 0.007), well below the recommended threshold of 0.01 [41]. This confirms equivalent factor loadings across groups, permitting meaningful comparisons of structural relationships.

#### 3.4.2. Structural Model Comparison

The unconstrained multi-group structural model yielded acceptable fit indices: RMSEA = 0.044 (90% CI [0.043, 0.045]), CFI = 0.906, TLI = 0.906, SRMR = 0.057. Then, a chi-square difference test comparing the unconstrained and constrained models revealed significant between-group heterogeneity in path coefficients (Δχ^2^(28) = 45.3, *p* < 0.05), supporting the need for BMI-specific models.

### 3.5. BMI-Specific Path Coefficients

The structural relationships among social-ecological factors, psychological factors, and PA differed markedly across BMI groups, revealing distinct patterns of influence (see Table 5).

Underweight Group (*n* = 187): For underweight adolescents, family support emerged as the primary driver of psychological readiness, significantly predicting stage of change (β = 0.290, *p* = 0.015), perceived benefits (β = 0.273, *p* = 0.027), and self-efficacy (β = 0.289, *p* = 0.015). In contrast, friend support had a narrower scope, predicting only self-efficacy (β = 0.280, *p* = 0.020). Notably, perceived barriers in this group did not respond to any socioecological factors. Moreover, the stage of change emerged as the only direct predictor of PA (β = 0.267, *p* = 0.001).

Normal Weight Group (*n* = 1070): This group exhibited the most robust and complex network of influences. PA was directly predicted by a combination of cognitive, social, and motivational factors: stage of change (β = 0.213, *p* < 0.001), perceived benefits (β = 0.281, *p* < 0.001), and friend support (β = 0.125, *p* = 0.001). Family support indirectly influenced PA via psychological mediators, reinforcing the multi-layered structure of behavior change among normal-weight youth.

Overweight and Obese Group (*n* = 316): This group presented a significant “socially dependent” pattern. Friend support (β = 0.136, *p* < 0.05) and the stage of change (β = 0.214, *p* < 0.01) jointly emerged as key direct predictors of PA levels, while internal cognitive factors (self-efficacy, perceived benefits) showed no direct influence. The socio-ecological pathway effect was particularly significant: the physical environment showed the strongest observed influence on perceived benefits across all groups (β = 0.471, *p* < 0.01). Similarly, friend support, as a dominant psychological resource, significantly enhanced self-efficacy (β = 0.370, *p* < 0.01), while effectively mitigating perceived barriers (β = −0.165, *p* < 0.05).

### 3.6. Structural Model Testing for Indirect Relationships Based on Groups

The researchers further analyzed the indirect effects within each group of the model. As shown in Table 6, the indirect effect analysis showed clear group differences.

Underweight Group: Mediating effects were limited. Only family support demonstrated a significant indirect effect on PA through stage change (β = 0.078, *p* < 0.05). Other indirect pathways involving friend support or environment were non-significant, confirming that family support is the primary driver of behavioral intent in this group.

Normal Weight Group: Displayed the most complex mediation network. Six indirect pathways were identified, confirming that socio-ecological factors (family, friends, and environment) influence PA by enhancing psychological readiness—specifically through the stage of change and self-efficacy. This reflects a “fully functional” system where external resources effectively translate into internal motivation and subsequent action.

Overweight/Obese Group: Crucially, no significant indirect effects were found. This “mediation disruption” indicates that while external factors (like peer support) may enhance self-efficacy (as shown in the structural model), this psychological capital does not automatically translate into behavioral engagement. highlights the need for direct external facilitation rather than relying solely on cognitive change.

## 4. Discussion and Implications

This study provides novel insights into BMI-specific pathways linking social-ecological and psychological factors with PA in Chinese adolescents. Our results essentially confirmed the proposed research hypotheses. Consistent with Hypothesis 1, we observed significant heterogeneity in both PA levels and psychosocial determinants across weight groups. Furthermore, in support of Hypothesis 2, the multi-group structural equation modeling revealed that the mechanisms predicting PA participation are structurally distinct across groups. By integrating the Social-Ecological Model with the Transtheoretical Model, our findings challenge the prevailing “one-size-fits-all” intervention approach. We demonstrate that while normal-weight adolescents operate on a comprehensive “dual-pathway” model—where environmental and social resources successfully translate into psychological readiness and subsequent action—this mechanism is structurally distinct for underweight and overweight/obese adolescents.

Our integrated framework successfully captured the complexity of PA determinants. The refined model achieved acceptable fit indices across the whole sample (CFI = 0.925, RMSEA = 0.039), validating the complementary value of combining ecological and psychological perspectives. This approach extends previous single-theory applications by revealing how environmental and social factors operate through stage-based mechanisms [42]. Our findings align with recent meta-analyses demonstrating that multi-level models explain significantly more variance in youth PA than single-level approaches [43,44,45]. Furthermore, the framework’s applicability to Chinese youth addresses calls by Sallis et al. for testing Western-developed theories in diverse cultural contexts [46].

Social support emerged as a key but BMI-dependent influence. Among normal-weight and underweight adolescents, family support acted as a primary antecedent, significantly shaping psychological factors such as stage of change, self-efficacy, and perceived benefits. However, family support did not directly predict PA behavior in these groups, suggesting its influence is fully mediated by individual psychological readiness. This highlights the “gatekeeper” role of parents in the Chinese context, where family expectations fundamentally shape adolescents’ cognitive orientation toward health behaviors [6,14].

In contrast, overweight and obese adolescents presented a very different pattern. For this group, friend support was a significant direct predictor of PA levels (β = 0.136) and the strongest predictor of self-efficacy (β = 0.370). More importantly, friend support is negatively correlated with perceived barriers (β = −0.165). This finding diverges from certain Western studies suggesting that “the peer environment is the primary source of weight stigma” [47]. Instead, it supports the “buffering hypothesis”—that for overweight Chinese adolescents, peer acceptance serves a protective function: it reduces psychological barriers while providing essential social facilitation, translating intentions into action [12,13,22]. The missing influence of family in this group may reflect developmental transitions during adolescence or underlying conflicts with family pressure related to weight, making peer acceptance a more potent behavioral driver.

The influence of the physical environment also varied dramatically. While the environment directly impacted psychological mediating variables in the normal-weight group, its effects on overweight and obese adolescents showed clear differences. Within this group, the physical environment produced the strongest observed effect on perceived benefits (β = 0.471), yet failed to directly predict PA participation. This indicates that while high-quality facilities significantly enhance overweight adolescents’ positive evaluations of exercise [48], this cognitive recognition does not automatically translate into actual participation. This disconnect reveals a unique intention-behavior gap among adolescents with high BMI—structural resources, though necessary, are insufficient to overcome behavioral habituation [49]. Transformation requires complementary social facilitation factors, such as peer support.

The stage of change consistently predicted PA levels across all groups, confirming its cross-cultural applicability [50]. However, differences emerged in mediating mechanisms: For normal-weight adolescents, the model revealed a functional mediation chain—external resources influenced PA through multiple psychological pathways (self-efficacy, perceived benefits, social support). Conversely, no significant indirect effects were observed among overweight and obese adolescents [51]. This phenomenon of “mediation disruption” suggests that increasing knowledge (benefits) or confidence (efficacy) alone may not motivate this group to engage in PA unless accompanied by direct external support [52,53]. For underweight adolescents, pathways were primarily driven by the stage of change and self-efficacy, with family support being the single distal influence factor [6]. This indicates that this group relies more on internal motivation and family support rather than broader environmental factors.

These findings suggest that PA interventions should move beyond uniform public health strategies toward more targeted, BMI-sensitive approaches. Rather than adopting a uniform intervention model, practitioners should tailor strategies to promote PA based on the unique motivations and psychosocial structures of different nutritional status groups. For overweight and obese adolescents, interventions may benefit from emphasizing peer-facilitated environments, such as group sports activities, to actively address psychological barriers and bridge the gap between intention and behavior. In contrast, strategies for underweight adolescents require a family-centered approach, reframing PA as essential for growth and development rather than weight management to engage parental support and foster internalized efficacy. Meanwhile, normal-weight adolescents may benefit most from integrated, multi-level interventions that combine environmental and psychological components. Collectively, these findings underscore the importance of a differentiated intervention framework designed to optimize public health resource allocation and enhance the effectiveness of PA promotion measures for diverse adolescent populations.

## 5. Limitations

This study integrates socioecological and psychological perspectives to examine specific patterns of PA participation among adolescents at different BMI levels, thereby highlighting the unique needs of underweight and overweight/obese youth and offering new insights to the literature. Despite these contributions, several limitations should be acknowledged when interpreting the findings. First, the cross-sectional design limits causal inferences about the observed associations. Second, data were collected from a single Chinese city, potentially limiting the generalizability of findings to other regions or cultural contexts. Third, PA was assessed via a self-report questionnaire, which may introduce recall bias despite the use of validated instruments. In addition, limited sample sizes in the underweight (*n* = 187) and overweight/obese (*n* = 316) groups prevented gender-stratified analyses required for Structural Equation Modeling, thereby restricting the examination of potential gender differences. Finally, overweight and obese adolescents were grouped for statistical stability; while this enhanced the reliability of estimates, it may mask subtle differences among overweight and obese adolescents. Future research should employ longitudinal designs, larger and more balanced samples, and objective PA measures (e.g., accelerometers), while differentiating between overweight and obese categories, to further validate and refine the proposed pathway mechanisms.

## 6. Conclusions

This study demonstrates that factors influencing adolescent PA vary across different BMI levels. Normal-weight adolescents benefit from a multi-level integrated approach, while underweight adolescents rely highly on family support and internal readiness. Overweight and obese adolescents show a “socially dependent” pattern, where friend support is crucial for overcoming barriers. These findings challenge one-size-fits-all intervention models, emphasizing that effective interventions must be structurally tailored: family education should be prioritized for underweight adolescents, while peer-promoting environments are needed for those with excess weight. By clarifying these BMI-specific pathways, this study provides an evidence-based foundation for the Healthy China 2030 plan, advocating for precision strategies targeting Chinese adolescents at both ends of the nutritional spectrum to meet their unique motivational needs.

## Figures and Tables

**Table 1:** Descriptive statistics of demographic characteristics and study variables of participants (*n* = 1573).

Characteristics	Frequency	Percentage (%)	Mean (SD)
Gender			
Boys	894	56.8%	
Girls	679	43.2%	
Age (year)			12 (1.68)
Weight (kg)			44.85 (11.39)
Height (cm)			154 (12)
BMI			18.75 (3.2)
Underweight	187	11.9%	15.6 (2.57)
Normal	1070	68%	17.93 (1.85)
Overweight/Obese	316	20.1%	23.38 (2.41)
Education Level			
Primary school	842	53.5%	
Middle school	731	46.5%	
Frequency of Weekly Exercise			4 (2.52)
Duration of Each Exercise			36.1 (31.1) *
PA (IPAQ)			
Low	335	31.3%	
Moderate	931	49.2%	
High	307	19.5%	

Notes: Values are presented as *n* (%) for categorical variables and mean (standard deviation, SD) for continuous variables; BMI = body mass index; PA = physical activity; IPAQ = International Physical Activity Questionnaire. Underweight, normal weight, and overweight/obese groups were classified based on age- and sex-specific BMI-for-age cut-offs. * Duration of each exercise is reported in minutes.

**Table 2:** Summary of measurement model fit indices.

Measurement Model	RMSEA (90% CI)	CFI	TLI	SRMR	Cronbach’s Alpha
SE-C	0.055 (0.052, 0.058)	0.931	0.924	0.040	0.966
PE-C	0.054 (0.034, 0.077)	0.996	0.989	0.010	0.886
DB-C	0.052 (0.044, 0.059)	0.973	0.964	0.036	0.809
SEE-C	0.066 (0.062, 0.070)	0.918	0.905	0.043	0.964

Note: RMSEA = Root Mean Square Error of Approximation; CFI = Comparative Fit Index; TLI = Tucker–Lewis Index; SRMR = Standardized Root Mean Square Residual. SE-C = Chinese version of Social Support for Exercise Scale; PE-C = Chinese version of Physical Environment for Exercise Scale; DB-C = Chinese version of Decisional Balance Scale; SEE-C = Chinese version of Self-efficacy for Exercise Scale. Cronbach’s alpha indicates internal consistency reliability.

**Table 3:** Model fit indices of the baseline model.

Model	RMSEA (90% CI)	CFI	TLI	SRMR
Acceptable threshold	<0.080	>0.920	>0.920	<0.080
Initial model	0.042 (0.041, 0.043)	0.907	0.903	0.050
Modified model	0.037 (0.036, 0.039)	0.925	0.921	0.049

Notes: RMSEA = Root Mean Square Error of Approximation; CFI = Comparative Fit Index; TLI = Tucker–Lewis Index; SRMR = Standardized Root Mean Square Residual. Acceptable threshold values were based on commonly used SEM criteria.

**Table 4:** Hypothesized path relationships in the baseline model.

Hypothesis	Pathways	β (95% CI) Standardized Path Coefficients	*p*-Value
H1a	FM → SOC	0.247 (0.167, 0.327)	<0.001
H1b	FM → BF	0.251 (0.168, 0.334)	<0.001
H1d	FM → SEE	0.226 (0.148, 0.305)	<0.001
H2a	FR → SOC	0.116 (0.040, 0.192)	=0.003
H2b	FR → BF	0.207 (0.127, 0.289)	<0.001
H2c	FR → BR	−0.168 (0.106, 0.229)	<0.001
H2d	FR → SEE	0.343 (0.269, 0.417)	<0.001
H3a	PE → SOC	0.097 (0.042, 0.153)	<0.001
H3b	PE → BF	0.349 (0.288, 0.409)	=0.001
H3c	PE → BR	−0.069 (−0.125, −0.013)	=0.015
H3d	PE → SEE	0.148 (0.092, 0.203)	<0.001
H4	SOC → PA	0.219 (0.166, 0.272)	<0.001
H5	BF → PA	0.089 (0.029, 0.150)	=0.004
H7	SEE → PA	0.104 (0.040, 0.168)	=0.001
H9	FR → PA	0.120 (0.061, 0.180)	<0.001
HA1	SEE7 ↔ SEE6	0.539 (0.473, 0.604)	<0.001
HA2	SEE6 ↔ SEE5	0.249 (0.171, 0.327)	<0.001
HA3	SEE8 ↔ SEE4	0.411 (0.342, 0.480)	<0.001
HA4	FM4 ↔FM1	0.426 (0.365, 0.487)	<0.001

Notes: β = regression weights of pathways. HA = additional path added to the model, FM = family support, FR = friends support, PE = physical environment for exercise, BF = perceived benefits, BR perceived barriers, SOC = Stage of change, SEE = Self-efficacy for exercise, PA = amount of physical activity. All path coefficients were estimated using structural equation modeling with robust maximum likelihood estimation.

**Table 5:** Standardized Path Coefficients Across BMI Groups.

Hypothesis	Underweight β (95% CI)	Normal β (95% CI)	Overweight/Obese β (95% CI)
FM → SOC	0.288 * (0.057, 0.520)	0.228 ** (0.136, 0.319)	0.287 * (0.078, 0.495)
FM → BF	0.268 * (0.027, 0.509)	0.278 ** (0.178, 0.377)	0.161 (−0.011, 0.333)
FM → SEE	0.270 * (0.046, 0.494)	0.232 ** (0.141, 0.323)	0.182 (−0.017, 0.381)
FR → SOC	0.169 * (−0.054, 0.392)	0.109 * (0.019, 0.199)	0.104 (−0.080, 0.288)
FR → BF	0.260 (−0.014, 0.535)	−0.184 ** (0.094, 0.274)	0.243 * (0.073, 0.412)
FR → BR	0.153 (−0.014, 0.320)	−0.169 ** (−0.244, −0.094)	−0.165 * (−0.298, −0.032)
FR → SEE	0.300 * (0.081, 0.519)	0.341 ** (0.252, 0.430)	0.370 ** (0.194, 0.546)
PE → SOC	0.189 * (0.041, 0.337)	0.081 * (0.016, 0.147)	0.094 (−0.043, 0.231)
PE → BF	0.283 * (0.114, 0.451)	0.321 ** (0.247, 0.395)	0.471 ** (0.349, 0.592)
PE → BR	−0.169 (−0.339, 0.001)	−0.075 * (−0.142, −0.008)	−0.007 (−0.118, 0.133)
PE → SEE	0.191 * (0.050, 0.332)	0.123 * (0.054, 0.193)	0.208 ** (0.092, 0.324)
SOC → PA	0.270 * (0.113, 0.426)	0.211 ** (0.146, 0.277)	0.214 ** (0.103, 0.326)
SEE → PA	0.164 * (0.005, 0.322)	0.092 * (0.016, 0.169)	0.093 (−0.065, 0.251)
BF → PA	0.056 (−0.120, 0.233)	0.095 * (0.022, 0.169)	0.103 (−0.034, 0.240)
FR → PA	0.096 (−0.087, 0.279)	0.120 * (0.048, 0.192)	0.136 * (0.008, 0.265)

Notes: β = standardized path coefficient; CI = confidence interval; FM = family support; FR = friend support; PE = physical environment for exercise; BF = perceived benefits; BR = perceived barriers; SOC = stage of change; SEE = self-efficacy for exercise; PA = physical activity amount. ** *p* < 0.01, * *p* < 0.05.

**Table 6:** The standardized indirect and total effects on PA amount across each group.

Group	Predictor Variables	Through	Causal Effect	
			Indirect β (*p*-Value)	Total β (*p*-Value)
Underweight	FM to PA:			0.122 * (0.012)
	FM	SOC	0.078 * (0.034)	
		SEE	0.044 (0.140)	
	FR to PA:			0.095 * (0.040)
	FR			
		SOC	0.046 (0.190)	
		SEE	0.049 (0.092)	
	PE to PA:			0.095 * (0.014)
	PE			
		SOC	0.051 (0.055)	
		SEE	0.031 (0.075)	
Normal Weight	FM to PA:			0.070 ** (<0.001)
	FM			
		SOC	0.048 ** (<0.001)	
		SEE	0.021 * (0.030)	
	FR to PA:			0.054 * (0.003)
	FR	SOC	0.023 * (0.024)	
		SEE	0.031 * (0.023)	
	PE to PA:			0.029 * (0.007)
	PE	SOC	0.017 * (0.027)	
		SEE	0.011 (0.056)	
Overweight/Obese	FM to PA:			0.078 * (0.019)
	FM			
		SOC	0.061 (0.026)	
		SEE	0.017 (0.332)	
	FR to PA:			0.057 (0.135)
	FR	SOC	0.022 (0.280)	
		SEE	0.034 (0.276)	
	PE to PA:			0.039 (0.082)
	PE	SOC	0.020 (0.223)	
		SEE	0.019 (0.256)	

Notes: Standardized indirect and total effects were estimated using bootstrapping procedures. FM = Family support, FR = Friend support, PA = Physical Activity amount, SOC = Stage of change, SEE = Self-Efficacy. Indirect effects were considered statistically significant when the *p*-value was <0.05. ** *p* < 0.01, * *p* < 0.05.

## Data Availability

The original contributions presented in the study are included in the article, further inquiries can be directed to the corresponding author.
